# 

**Published:** 1821-06

**Authors:** 


THE LONDON
### Medical and Physical Journal.

6 OF VOL. XLV.] JUNE, 1821. [NO. 268.

### NOTICE.

*Another of the series of* PRÖEMIA *to the several volumes of this Journal, (which commenced with that to the forty-third volume,) comprising a History of the Progress of Medicine and its auxiliary Sciences for the half-year immediately previous to the period of their production, respectively, will be published on the last day of July. One of the especial intentions of those* Pröemia, *is to present a comprehensive view of the state and progress of Medicine throughout Europe generally, and in the United States of America; an object that cannot be effected in the regular monthly Numbers of the Journal, because of the small extent of space which can be there appropriated to this purpose.*

### MONTHLY CATALOGUE OF MEDICAL BOOKS.

Address to the Public, relative to some supposed Failures of the Cow-pox, at Repton and its Neighbourhood; with Observations on the Efficacy and general Expediency of Vaccination, and on the Injurious Consequences of Inoculation for the Small-pox. By B. Granger, Surgeon. 8vo.

A Treatise on Gonorrhœa and Syphilis. 12mo. 4s. 6d.

A Manual of Chemistry. By W. T. Brande. 3 vols.

A Treatise on Gun-shot Wounds, on Injuries of the Nerves, and on the Wounds of the Extremities. With five explanatory Plates. By G. J. Guthrie.

A Treatise on Indigestion and its Consequences, called Nervous and Bilious Complaints; with Observations on the Organic Diseases in which they sometimes terminate. By A. P. W. Phillip, M.D. F.R.S. Edin.

[HIGHLEY AND SON, FLEET-STREET.]

